# Multiple Stem/Progenitor Cells Isolated from the Limbus

**DOI:** 10.2174/011574888X358606250414063844

**Published:** 2025-04-29

**Authors:** Xuying Wang, Guigang Li

**Affiliations:** 1 Department of Ophthalmology, Tongji Hospital, Tongji Medical College, Huazhong University of Science and Technology, Wuhan, Hubei Province, 430030, China

**Keywords:** Cornea, limbus, limbal epithelial stem cell, limbal niche cell, limbal melanocyte, stem cell niche, limbal stem cell deficiency

## Abstract

Limbal epithelial stem cells (LESCs), which are responsible for the renewal and repair of corneal epithelium, are located in the limbus. The limbus is an important structure for maintaining the normal corneal epithelium. Damage to the limbus can lead to limbal stem cell deficiency (LSCD), a common blind-causing disease. However, the cellular composition of the limbus and the functions of various cell populations have not yet been accurately reproduced, making it difficult to reconstruct the normal structure of the limbus under disease conditions. Currently, there are mature methods for isolating and culturing various types of stem/progenitor cells from the limbus, including LESCs, limbal niche cells (LNCs), and limbal melanocytes (LMs). Successful culture of these cells helps to better investigate their biological functions, their role in sustaining corneal epithelial homeostasis, and their feasibility for basic research or clinical applications. This review summarizes the definitions, functions, and characteristics of these three types of stem/progenitor cells that can be isolated and purified from the limbus, in the hope of drawing attention to and stimulating discussion on this topic. This will help to clarify the cellular composition of the limbus, reconstruct the normal structure of the limbus, and develop innovative stem cell therapy.

## 1 INTRODUCTION

Stem cells are a group of cells with the potential for self-renewal and monodirectional or multidirectional differentiation. They are capable of self-replication and, under certain conditions, can also differentiate into a variety of different tissue cells to form various tissues and organs in the human body [1, 2]. According to the differentiation potential of stem cells, the level of differentiation, and their functions, they can be roughly divided into three types: embryonic stem cells, tissue stem cells, and specialized stem cells. Embryonic stem cells, also known as totipotent stem cells, are isolated and cultured from the early embryos of mammals, including human beings, from which complete biological individuals can be formed. Upon further differentiation, embryonic stem cells can form a variety of tissue stem cells, also known as pluripotent stem cells, which have the potential to differentiate into a variety of cells and tissues. Under specific conditions, pluripotent stem cells can differentiate into specialized stem cells or progenitor cells, which can only differentiate into a certain type of cells [3]. Adult stem cells in many differentiated tissues are typically unipotent stem cells that can only give rise to one cell type under normal circumstances [4]. Adult stem cells are relatively scarce undifferentiated cells in many organs and differentiated tissues, with limited self-renewal [5, 6] and differentiation capabilities [7]. These cells have a promising clinical application as seed cells for use in cell therapy with tissue and organ replacement therapy [8, 9]. The advancement of knowledge regarding stem cells has given rise to a corresponding interest in their surroundings. The concept of stem cell niche was first introduced in 1978 and refers to the specific microenvironment in which adult stem cells reside in their tissues [10]. Detachment from the stem cell niche or disruption of the stem cell niche results in the loss of stemness of stem cells [11]. It is generally accepted that ecological niches, which include cytokines, extracellular matrix, and other cells, act synergistically and collectively participate in maintaining the phenotype of undifferentiated stem cells [6, 12].

The corneal epithelium is a self-renewable tissue, and by definition, it is governed by stem cells [13]. Previously, it was thought that the basal layer of central corneal epithelial cells had stem cell potential because they could continuously produce mature corneal epithelial cells. In 2008, Majo *et al*. proposed that the corneal epithelium contains stem cells because it is capable of generating individual colonies and that the central corneal epithelium in mice can be transplanted and regenerated continuously. These findings seem to support the idea that the corneal epithelium itself contains stem cells [14]. However, extensive experimental and clinical evidence has shown that this population of stem cells is not in the corneal epithelium but concentrated in the limbal basal epithelium [15]. For instance, experiments have shown that central corneal epithelial cells uniformly express K3 (keratin 3), which marks late differentiation of corneal epithelial cells, and this uniform expression suggests that cells in the basal layer of central corneal epithelium are more differentiated than limbal epithelial basal cells that do not express K3 [7]. One of the typical manifestations of epithelial stem cells *in vivo* is relatively rare proliferation, and such cells were observed to be confined to the basal layer of the limbal epithelium [16]. The size of human limbal and central corneal basal epithelial cells was measured by confocal microscopy and flow cytometry; the smallest cells were located in the limbal basal epithelium [17]. LESCs exhibited greater proliferative capacity than central corneal epithelial cells during *in vitro* culture experiments [18]. Although progress has been made in the culture and expansion techniques of LESCs, there are still many challenges to overcome. For example, identifying LESCs, maintaining their stemness, and improving cell expansion efficiency are all significant issues. These challenges have led to a greater emphasis on the study of the limbus, including cellular composition, extracellular matrix, and their interactions, in the hope of finding solutions that will lead to greater breakthroughs in the clinical application of LESCs.

The limbus is the transitional zone between the cornea and conjunctiva where Vogt palisades exist [19]. In this structure, the epithelium increases in number of layers and extends down into the stroma, while the stroma extends into the epithelium in finger-like projections, forming a fenestrated epithelial-stromal staggered organization [20]. Since LESCs are present in the limbus, this area is called the LESCs niche [21]. In this niche, the association between LESCs and supporting cell populations, including LNCs and LMs, constitutes an important factor in maintaining stem cell potency [22]. Co-culturing these cells *in vitro* supports LESCs growth and preserves their stem cell characteristics [23]. Given the interest in the biological functions of ecological niche cell populations, suitable methods are available to isolate and expand LNCs and LMs from limbus to enable them to self-renew and produce more cells *in vitro* [24, 25]. Isolated and cultured LNCs have characteristics of mesenchymal stem cells (MSCs) and can be induced to develop into corneal epithelial cells and corneal stromal cells, among others, and express embryonic stem cell markers and vascular progenitor cell markers. Isolated and cultured LMs were able to self-renew and continuously produce and secrete melanin. When seeded at low density in Petri dishes *in vitro*, LMs can form colonies, suggesting a melanocyte progenitor cell phenotype. Fig. (**1**) is a schematic diagram of the limbus, illustrating the renewal process of the corneal epithelium and the positional relationships among LESCs, LNCs, and LMs.

## 2 LIMBAL EPITHELIAL STEM CELLS (LESCs)

LESCs are located in the basal layer of the limbal epithelium and are a population of self-renewing unipotent stem cells [2, 7]. The concept that the human corneal epithelium is maintained by LESCs residing in the limbus has been recognized for 30 years [16]. In normal physiology, LESCs proliferate slowly; when the corneal epithelium is damaged, the proliferative capacity of LESCs is enhanced [16, 26]. LESCs can divide symmetrically, giving rise to two identical daughter stem cells, or asymmetrically, giving rise to a daughter stem cell that maintains its original proliferative capacity and a transiently amplifying cell that has a limited ability to proliferate [27]. Transient amplifying cells (TACs) proliferate and migrate toward the central cornea, which then differentiates into terminally differentiated cells (TDCs), which migrate upward and eventually detach from the corneal surface due to normal functions such as blinking [28]. The X/Y/Z hypothesis, proposed by Thoft *et al*., on the process of LESCs division is well recognized [29]. Due to the lack of specific molecular markers, a direct method for recognizing LESCs has not been established, although multiple LESCs-related markers have been proposed. New markers are constantly being proposed [30-32]. Presently, validated markers for LESCs can be classified into at least three categories: 1) nuclear proteins: One widely used marker is p63. A member of the p53 gene family, p63 has been implicated in stem cell regulation as a factor that maintains cell cycle arrest and genome stability [33]. p63 has been immunodetected predominantly in the nuclei of certain limbal epithelial basal cells [26]. 2) cytoplasmic proteins: Cytokeratin (K) is differentially expressed in corneal epithelial cells and can be used to recognize the degree of differentiation of corneal epithelial cells. LESCs specifically express K5 and K14 and do not express K3 and K12 [34]. 3) cell membrane or transmembrane proteins: ABCG2 (ATP Binding Cassette Subfamily G Member 2) [35] and ABCB5 (ATP-binding cassette, sub-family B, member 5) [36] are expressed on the cell surface in some limbal basal epithelial cells. Integrin α9 is immunodetected in the cell membrane and cytoplasm of some limbal basal epithelial cells, but not in cells above the limbal epithelial base or in keratocytes. This is similar to the expression pattern of p63 and ABCG2 [26]. Moreover, the absence of gap junction components is thought to be a characteristic of stem cells [37]. E-cadherin and connexin 43 were only observed in the cornea and above the basal epithelium of the limbus [26].

LESCs isolated from the basal epithelium of the limbus show heterogeneity. Studies using single-cell transcriptomics to identify limbal cell populations and cell types have found that nine cell clusters are annotated as LESCs and their progeny, including terminally differentiated cells (TDCs), post-mitotic cells (PMCs), transient amplifying cells (TACs), limbal epithelial progenitor cells (LEPCs), and LESCs [28]. These cell clusters are distinguished based on corneal epithelium-specific differentiation markers, keratinization markers, major proliferation markers, and stem cell markers. Traditionally, LESCs have been cultured in media containing xenogeneic components, such as fetal bovine serum [38] and mouse 3T3 feeder cells [39]. Plastic culture dishes [40] and human amniotic membrane [41] are the commonly used as growth substrates in traditional LESCs culture systems. Over the past few decades, significant advancements and innovations in LESCs’ culture techniques have been made to ensure higher biosafety and lower immunogenicity in further cell processing. These advancements include the complete removal of xenogeneic components from the culture media [42], the application of human feeder cells [38], and the development of novel scaffolds [43], thereby achieving better cell growth and proliferation.

LESCs are protected within the limbus [27]. Papillae form at the base of the limbal epithelium and become specialized fenestrated epithelial structures called limbal palisades of Vogt, which contain pigment and a rich vascular network and are in close contact with the basement membrane [20, 44]. In addition to the presence of LESCs, the LESCs niche is home to a variety of supporting cells, an extracellular matrix with specific characteristics, and soluble factors that facilitate the transmission of biochemical and biophysical signals [45]. The limbal epithelium is thicker and has more cell layers than the corneal epithelium [46]. The limbal region contains abundant blood vessels, which provide LESCs with sufficient nutrients and growth factors [47]. The well-developed nerves in the limbal region provide LESCs with the neurotrophic factors needed to maintain growth [48]. The function of LESCs to naturally replace corneal epithelium and heal corneal epithelial injuries is dependent on the coordinated interaction of multiple factors within the LESCs niche [23]. LESCs and their microenvironment are a harmonious whole, and damage to any one of them may lead to limbal stem cell deficiency (LSCD). LSCD is a corneal epithelial homeostatic imbalance ocular surface disease caused by a decrease in the number or function of LESCs. LSCD is manifested by persistent epithelial defects and conjunctivization of the corneal surface, which can lead to visual impairment and chronic ocular surface pain [49]. Partial or total loss of LESCs results in impaired renewal and healing of the corneal epithelium, which leads to remodeling of the ocular surface due to bulbar conjunctival invasion, referred to as ‘conjunctivization’ of the cornea [50]. In recent decades, people have explored many methods for treating LSCD, which are essentially based on restoring the LESCs’ cell pool [51, 52] and reconstruction of the microenvironment [53]. LESCs obtained by isolation from the limbus can produce long-term proliferating clones *in vitro*, which can be used for corneal epithelial regeneration in patients with severe damage to the ocular surface due to injury or disease [54-56]. Despite the progress of LESCs-based cell transplantation in the treatment of LSCD, it still faces many challenges, including limited cell sources, limitations in *in vitro* culture techniques, immune rejection and immunosuppression.

## 3 LIMBAL NICHE CELLS (LNCs)

LNCs are a kind of pluripotent stem cells from the limbal epithelium subjacent and stroma of the limbus, with the characteristics of mesenchymal stem cells (MSCs), discovered in recent years [57]. Different researchers have used different isolation and culture methods and named them differently, such as limbal niche cells [24, 58], corneal stromal stem cells [59], peripheral and limbal corneal stromal cells [60], limbal biopsy-derived stromal cells [61], limbal mesenchymal stem cells [62], and limbal fibroblasts [63], *etc*. The common characteristics of these cells are that they all originate from the limbal stroma, have the function of MSCs, can be induced to differentiate into myofibroblasts, corneal epithelial cells, corneal stromal cells, *etc*., and are capable of supporting LESCs under *in vitro* culture conditions [64, 65]. They are uniformly referred to as LNCs in this review. LNCs are usually considered as LESC-regulating mesenchymal stem cells [66]. LNCs express VIM (vimentin) and do not express PCK (pan-cytokeratin). VIM is a type III intermediate filament protein that anchors and supports organelles within the cytoplasm of mesenchymal cells. VIM is used as a marker for cells of mesenchymal origin or cells undergoing epithelial-mesenchymal transition. Cytokeratin is mainly distributed in epithelial cells and its main function is to maintain the integrity and continuity of epithelial tissue [67]. LNCs express the embryonic stem cell markers Oct4 (octamer-binding transcription factor), Sox2 (sex determining region Y-box 2), Rex1 (reduced expression1), SSEA4 (stage-specific embryonic antigen 4), Nanog, Nestin, N-Cadherin; MSC surface markers CD73, CD90, and CD105; and vascular progenitor cell markers such as CD34, PDGFRβ (platelet-derived growth factor receptor β) [57, 58, 64]. Compared to bone marrow mesenchymal stem cells (BMMSC), LNCs express more embryonic stem cell markers (*e.g*., Nestin, Rex1, and SSEA4), MSCs markers (*e.g*., CD73, CD105), neural crest cell markers (*e.g*., MSX1 (muscle segment homeobox gene 1), p75NTR (p75 neurotrophin receptor)), vascular progenitor cell markers, PDGFRβ, and SCF (stem cells factor). These findings indicate that the LNCs have stronger stem cell properties than BMMSCs and are an important source of stem cells in the limbus [68].

LNCs are located primarily in the anterior stroma, near the border of the limbal epithelium. Their localization allows them to play a role in supporting and maintaining LESCs [61, 69]. It has been demonstrated that the co-culturing of LESCs with LNCs on 3D Matrigel *in vitro* results in their spherical growth. Furthermore, it was established that this co-culture prevents LESCs from differentiating into mature cells and maintains their stem cell status, as evidenced by decreased CK12 expression and elevated p63α expression, as well as increased efficiency of all-clone formation on the 3T3 feeder layer [57]. Moreover, when LNCs were co-cultured with mature corneal epithelial cells (MCECs), the expression of CK12 in MCECs was decreased and the expression of p63α was increased, suggesting that LNCs can induce dedifferentiation of MCECs [67]. Animal experiments demonstrated that subconjunctival transplantation of human LNCs or BMMSCs prevented LSCD in a rabbit model of alkali burns, and the results of these two were compared. The efficacy of both LNCs and BMMSCs in preventing LSCD was evidenced by a reduction in the area of epithelial defects, a reduction in corneal neovascularization, and a reduction in the number of goblet cells. Furthermore, the therapeutic efficacy of subconjunctival grafts was superior when using LNCs compared to BMMSCs [68]. In an animal model of corneal physical injury, it was found that LNCs could rebuild the corneal stroma while inhibiting inflammatory responses and reducing corneal stromal scarring [59, 70]. The mechanism by which LNCs promote scarless healing of the corneal stroma may involve their inhibition of neutrophil migration to the injured stroma, which inhibits the deposition of fibrotic tissue [71]. As microenvironmental cells of LESCs, LNCs can mimic the stem cell microenvironment *in vitro* and support the growth of LESCs; LNCs as therapeutic cells can promote the healing of corneal epithelium after transplantation to prevent and treat LSCD after corneal alkali burns; and LNCs can promote the healing of corneal stroma to reduce scarring and neovascularization, *etc*. Moreover, LNCs showed superior effects than BMMSCs in supporting LESCs and treating LSCD. Therefore, as limbus-derived progenitors for MSCs, LNCs may be more ideal seed cells for corneal disease stem cell therapy and tissue engineering than other sources of MSCs.

## 4 LIMBAL MELANOCYTES (LMs)

LMs are present in the limbal basal epithelium and possess the capacity for self-renewal and continuous production and secretion of melanin [72]. A recent comprehensive single-cell RNA sequencing study has identified the presence of CPVL (carboxypeptidase staphylococcal-like)^+^ cells in the limbal basal epithelium. Additionally, a subset of these cells has been observed to express ΔNp63 and are located near cells of LESCs that express K15. The cell cluster exhibited high expression of PAX6 (paired box 6). Pax6 is a transcription factor that plays a pivotal role in the regulation of eye and brain development. Furthermore, it helps maintain the phenotype of neural crest progenitors. It can therefore be proposed that this cluster represents limbal neural crest-derived progenitors (LNCPs). Furthermore, immunofluorescence analysis demonstrated a significant overlap in the expression of the neural crest marker MITF (microphthalmia-associated transcription factor) and the cluster-specific marker CPVL. This provides further evidence to support the conclusion that this cell population is of the LNCP nature [22]. LMs are located within clusters of basal epithelial cells and are closely associated with LESCs [73, 74]. The direct contact between the LMs and the LESCs appears to be mediated by cell adhesion molecules [75]. It is difficult to isolate and culture LMs *in vitro* because of their small number, low percentage in the total number of limbal cells, and poor proliferative capacity compared with LESCs and LNCs [76]. A contemporary approach to the efficient isolation of LMs employs the use of CD90 and CD117 as selection markers, facilitating the attainment of pure LMs through fluorescence-activated cell sorting. This sorted LMs population, characterized by the expression of CD117 and lack of CD90, exhibits the capacity for self-renewal and sustained melanin production. LMs cultured after sorting showed large, flattened, smooth bodies. Furthermore, when cultured *in vitro* at low density on medium, LMs demonstrated the capacity to form colonies, indicative of a melanocyte progenitor cell phenotype [25, 77]. The most commonly accepted markers of LMs are as follows, although this list is not exhaustive: The following markers are commonly used in the diagnosis of LMs: CD117 (c-Kit), Melan-A, tyrosinase-related protein 1 (TRP1), human melanoma black-45 (HMB45) and SRY-box transcription factor 10 Sox10 [72].

The direct contact between LMs and LESCs in the limbal epithelial basal layer demonstrates that LMs may help maintain stem cell properties of LESCs [72, 78]. The following points can be summarized about the role of LMs: 1) Protective role: LMs are capable of reducing UV-induced damage to LESCs by secreting and delivering melanin granules. This protective effect is primarily evidenced by the ability of limbal melanocytes to cover the nuclei of epithelial cells following UV-B irradiation, forming the so-called 'melanophore', which serves to reduce the direct damage to LESCs caused by UV-B [73, 79]. The study revealed that the highest levels of pigmentation were observed in the most primitive cells, indicating that the degree of pigmentation is correlated with the differentiation status of LESCs [80]. 2) Promote cell proliferation and stem cell protection: LMs not only protect LESCs from DNA damage and apoptosis [79] but also maintain the stemness of LESCs. Studies have shown that LMs promote corneal epithelial wound healing and the formation of multilayered epithelial cell sheets [75, 81]. 3) Anti-inflammatory and anti-angiogenic: LMs can effectively inhibit the proliferation and cytokine secretion of alloreactive T cells and possess potent anti-angiogenic properties [75]. In conclusion, LMs play a critical role in protecting LESCs from external damage, promoting cell proliferation, and maintaining stemness. These functions guarantee the normal physiological state of the cornea and the correct functioning of the visual system. Table **1** summarizes the current commonly accepted positive markers for the three cell types, LESCs, LNCs, and LMs, and the roles they play in the limbus.

## 5 DISCUSSION

The cornea is located in the anterior segment of the eyeball and has a unique transparent structure. The corneal epithelium is constantly replaced by new ones differentiated from LESCs. Currently, much evidence indicates that this population is located in the limbus and is referred to as LESCs. As a result of the emphasis on LESCs, there has been a surge in research on the microenvironment of the limbus, making its composition more clearly understood. It has been demonstrated that the co-culture of LESCs with LNCs is an effective method for maintaining the stem cell properties of LESCs [86-88]. LNCs have MSCs properties and are believed to possess potent immunomodulatory, anti-inflammatory, and anti-angiogenic capabilities, which have been substantiated by empirical evidence. This renders LNCs a highly promising avenue for clinical applications in the management of comorbid inflammatory conditions [59, 89]. Due to the low proportion of LMs in the limbal epithelial cells and their relatively low mitotic activity compared to other cell types, culturing LMs *in vitro* is quite challenging [90, 91]. The current solution involves leveraging the fact that primary LMs express CD117 on their cell surface [92, 93]. By using CD90^-^ and CD117^+^ as selection markers, highly purified primary LMs can be isolated through fluorescence-activated cell sorting (FACS) technology [77]. These isolated LMs are then cultured using recombinant LN-511-E8 as the culture substrate [72]. This method of isolation and culture results in LMs that possess the ability to self-renew and continuously produce melanin. However, LMs isolated from the limbus may also be heterogeneous [93]. LMs synthesize melanin to safeguard LSCs from UV-B radiation and to neutralize reactive oxygen species (ROS). Direct contact between LMs and LESCs suggests that LMs may contribute to maintaining the function of LESCs [73, 94]. Previous studies proved that LMs and LESCs can preserve the stem cell characteristics of LESCs when cultivated together *in vitro* [80]. Additionally, LMs have been shown to facilitate the regenerative process of the corneal epithelium during wound healing [81] and to enhance the growth of LESCs in tissue-engineered corneal epithelial constructs [72, 76].

A greater number of studies are now available for exploring the specific mechanisms of the interaction between LESCs and their supporting cells. LESCs and LNCs combined activate the CXCR4/SDF-1 chemokine axis, resulting in significantly increased sphere size, secondary colony formation efficiency, and expression of stem cell markers [87]. In an *in vitro* LESCs-LNCs co-culture system, LNCs inhibited the expression of Notch signaling in LESCs and upregulated their stemness [95, 96]. Additionally, LNCs controlled the clonal growth of LESCs through BMP signaling pathway as well as Wnt signaling pathway [97]. Moreover, the KIT/AKT signaling pathway has been demonstrated to play a key role in the interaction between LNCs and LESCs, and Sox10 expressed by LNCs stimulates the production of KIT ligand, thereby activating the KIT/AKT signaling pathway, which protects LESCs against caspase-3-associated cell death [98]. LMs can support LESCs through CD117/SCF signaling [68, 81]. These mechanisms could help to improve *in vitro* tissue engineering and *in vivo* LSCD therapy. Currently, research on the support of LMs for LESCs is limited to the cellular and molecular levels, while research on the support of LNCs for LESCs has entered the animal experiment stage. Future studies will need more evidence to explore the comparative efficacy of LNCs and LESCs in clinical settings.

The continuous proliferation and differentiation of LESCs are essential for the natural turnover of corneal epithelium in the normal state and for wound healing in the pathological state. The proliferation and associated properties of LESCs are contingent upon the microenvironment [99]. It is distinguished by a high degree of vascularization and innervation within the stroma [100]. Supporting cells, extracellular matrix, and soluble factors in the limbal niche are involved in the regulation of LESCs [101]. Impairment of LESCs or the LESCs niche results in a decrease in the number and function of LESCs [50, 102]. In mild partial LSCD, medications to improve the ocular surface may be sufficient, whereas in severe or total LSCD, LESCs need to be supplemented by transplantation of LESCs [103, 104]. Currently, the indication for direct autologous LESCs transplantation is limited to unilateral LSCD [105], and the limitation of this technique lies in the risk of inducing donor LSCD when harvesting the autologous limbal graft [106]. Bilateral LSCD requires allogeneic limbal grafts, but due to the high risk of rejection, the 5-year survival rate is only 50% [107]. Cultured limbal epithelial transplantation (CLET) represents a potential treatment for severe to complete LSCD [108]. Research demonstrated the viability and safety of CLETs manufactured under the xenobiotic-free condition in clinical therapy [109, 110]. Further research is underway to investigate alternative cell sources for corneal epithelial cell repopulation. The initial clinical trial using MSCs has yielded promising short-term results [111-113]. Despite the great potential of LESCs-based cell therapies for the treatment of LSCD, their large-scale clinical application remains limited. This is mainly attributed to the limited sources of cells, the difficulty in obtaining high numbers of cells for *in vitro* expansion, and the inability to guarantee the efficacy and safety of stem cells.

## CONCLUSION

In conclusion, at least three stem/progenitor cell types have been isolated and characterized from the limbus, including LESCs, LNCs, and LMs. These cells play crucial roles in maintaining the normal structure and function of the corneal epithelium. LESCs are responsible for the renewal and repair of the corneal epithelium, while LNCs and LMs support and regulate LESCs. Cell isolation and culture *in vitro*, exploration of cell signaling pathways, construction of organoids, and comprehensive single-cell RNA sequencing have provided a deeper understanding of the cellular composition and niche regulation of the limbus. These methods have paved new ways for the reconstruction of the limbus.

In the future, it may be possible to isolate and characterize new stem/progenitor cell types from this anatomical region. A greater focus on in-depth studies of LESCs and their microenvironment will facilitate a deeper understanding of the mechanisms underlying normal corneal epithelial renewal and corneal epithelial wound healing. This, in turn, will enable the exploration of safer and more effective clinical treatments for LSCD. In addition, interdisciplinary collaboration is important to advance the study of stem cells within limbus, and a variety of materials have been used in corneal regeneration and the treatment of LSCD [114, 115]. Reconstructing the limbus and restoring the normal microenvironment of LESCs are areas to focus on in the future, which will provide more possibilities for clinical treatment and scientific research.

## Figures and Tables

**Fig. (1):**
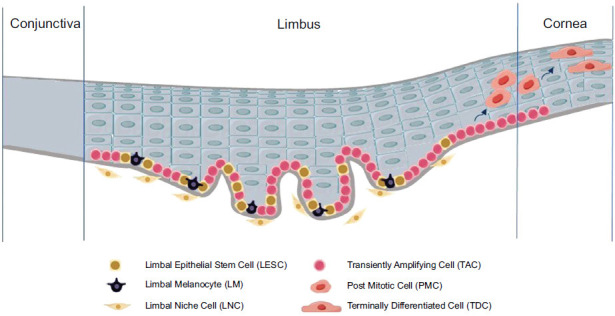
Overview of the structure of limbus. The style of the cells referenced corresponding materials from Figdraw (https://www.figdraw.com).

**Table 1:** Currently commonly accepted positive markers for LESCs, LNCs, and LMs, and their functions.

**Cell Type**	**Positive Makers**	**Functions**
Limbal Epithelial Stem Cells	p63 [82], ABCG2 [83], ABCB5 [36], CK5, CK14 [84], CK19 [85]	Under physiological conditions, continuously renew the corneal epithelium Under pathological conditions, repair the corneal epithelium
Limbal Niche Cells	VIM, CD73, CD90, CD105 [57], SCF [67], PDGFRβ^86^, CD34 [64]	Support and maintenance of LESCs As therapeutic cells for LSCD
Limbal Melanocytes	CD117, Melan-A, TRP1, HMB45, Sox10^25^	Mitigating UV damage to LESCs by secretion and delivery of melanin granules Maintaining the stemness of LESCs Anti-inflammatory and anti-angiogenic

## LIST OF ABBREVIATIONS

ABCB5: ATP Binding Cassette Subfamily B Member 5
ABCG2: ATP Binding Cassette Subfamily G Member 2
BMMSCs: Bone Marrow Mesenchymal Stem Cells
CPVL: Carboxypeptidase Staphylococcal-like
FACS: Fluorescence-activated Cell Sorting
HMB45: Human Melanoma Black-45
K3: Keratin 3
LEPCs: Limbal Epithelial Progenitor Cells
LESCs: Limbal Epithelial Stem Cells
LMs: Limbal Melanocytes
LNCPs: Limbal Neural Crest-derived Progenitors
LNCs: Limbal Niche Cells
LSCD: Limbal Stem Cell Deficiency
MCECs: Mature Corneal Epithelial Cells
MSCs: Mesenchymal Stem Cells
MSX1: Muscle Segment Homeobox Gene 1
Oct4: Octamer-Binding Transcription Factor
p75NTR: p75 Neurotrophin Receptor
PAX6: Paired Box 6
PCK: Pan-Cytokeratin
PDGFRβ: Platelet-Derived Growth Factor Receptor β
PMCs: Post-Mitotic Cells
Rex1: Reduced Expression 1
ROS: Reactive Oxygen Species
SCF: Stem Cells Factor
Sox10: SRY-Box Transcription Factor 10
Sox2: Sex Determining Region Y-Box 2
SSEA4: Stage-Specific Embryonic Antigen 4
TACs: Transient Amplifying Cells
TDCs: Terminally Differentiated Cells
TRP1: Tyrosinase-Related Protein 1
VIM: Vimentin

## References

[1] Leblond C.P. (1981). The life history of cells in renewing systems.. Am. J. Anat..

[2] Tseng S.C.G. (1989). Concept and application of limbal stem cells.. Eye (Lond.).

[3] Wagers A.J., Weissman I.L. (2004). Plasticity of adult stem cells.. Cell.

[4] Post Y., Clevers H. (2019). Defining Adult stem cell function at its simplest: The ability to replace lost cells through mitosis.. Cell Stem Cell.

[5] Fuchs E., Tumbar T., Guasch G. (2004). Socializing with the Neighbors.. Cell.

[6] Spradling A., Drummond-Barbosa D., Kai T. (2001). Stem cells find their niche.. Nature.

[7] Schermer A., Galvin S., Sun T.T. (1986). Differentiation-related expression of a major 64K corneal keratin in vivo and in culture suggests limbal location of corneal epithelial stem cells.. J. Cell Biol..

[8] Weissman I.L. (2000). Translating stem and progenitor cell biology to the clinic: Barriers and opportunities.. Science.

[9] De Luca M., Aiuti A., Cossu G., Parmar M., Pellegrini G., Robey P.G. (2019). Advances in stem cell research and therapeutic development.. Nat. Cell Biol..

[10] Schofield R. (1978). The relationship between the spleen colony-forming cell and the haemopoietic stem cell.. Blood Cells.

[11] Walther V., Graham T.A. (2014). Location, location, location! The reality of life for an intestinal stem cell in the crypt.. J. Pathol..

[12] Conti L., Pollard S.M., Gorba T. (2005). Niche-independent symmetrical self-renewal of a mammalian tissue stem cell.. PLoS Biol..

[13] Kumar A., Yun H., Funderburgh M.L., Du Y. (2022). Regenerative therapy for the Cornea.. Prog. Retin. Eye Res..

[14] Majo F., Rochat A., Nicolas M., Jaoudé G.A., Barrandon Y. (2008). Oligopotent stem cells are distributed throughout the mammalian ocular surface.. Nature.

[15] Sun T.T., Tseng S.C., Lavker R.M. (2010). Location of corneal epithelial stem cells.. Nature.

[16] Cotsarelis G., Cheng S.Z., Dong G., Sun T.T., Lavker R.M. (1989). Existence of slow-cycling limbal epithelial basal cells that can be preferentially stimulated to proliferate: Implications on epithelial stem cells.. Cell.

[17] Romano A.C., Espana E.M., Yoo S.H., Budak M.T., Wolosin J.M., Tseng S.C.G. (2003). Different cell sizes in human limbal and central corneal basal epithelia measured by confocal microscopy and flow cytometry.. Invest. Ophthalmol. Vis. Sci..

[18] Ebato B., Friend J., Thoft R.A. (1988). Comparison of limbal and peripheral human corneal epithelium in tissue culture.. Invest. Ophthalmol. Vis. Sci..

[19] Seyed-Safi A.G., Daniels J.T. (2020). The limbus: Structure and function.. Exp. Eye Res..

[20] Davanger M., Evensen A. (1971). Role of the pericorneal papillary structure in renewal of corneal epithelium.. Nature.

[21] Soleimani M., Cheraqpour K., Koganti R., Baharnoori S.M., Djalilian A.R. (2023). Concise review: Bioengineering of limbal stem cell niche.. Bioengineering.

[22] Collin J., Queen R., Zerti D. (2021). A single cell atlas of human cornea that defines its development, limbal progenitor cells and their interactions with the immune cells.. Ocul. Surf..

[23] Polisetti N., Zenkel M., Menzel-Severing J., Kruse F.E., Schlötzer-Schrehardt U. (2016). Cell adhesion molecules and stem cell-niche-interactions in the limbal stem cell niche.. Stem Cells.

[24] Su G, Wang W, Xu L (2023). Isolation and identification of limbal niche cells.. J Vis Exp.

[25] Polisetti N., Sharaf L., Schlötzer-Schrehardt U., Schlunck G., Reinhard T. (2022). Efficient isolation and functional characterization of niche cells from human Corneal Limbus.. Int. J. Mol. Sci..

[26] Chen Z., de Paiva C.S., Luo L., Kretzer F.L., Pflugfelder S.C., Li D.Q. (2004). Characterization of putative stem cell phenotype in human limbal epithelia.. Stem Cells.

[27] Stepp M.A., Zieske J.D. (2005). The corneal epithelial stem cell niche.. Ocul. Surf..

[28] Di Girolamo N. (2015). Moving epithelia: Tracking the fate of mammalian limbal epithelial stem cells.. Prog. Retin. Eye Res..

[29] Thoft R.A., Friend J. (1983). The X, Y, Z hypothesis of corneal epithelial maintenance.. Invest. Ophthalmol. Vis. Sci..

[30] Li D.Q., Kim S., Li J.M. (2021). Single-cell transcriptomics identifies limbal stem cell population and cell types mapping its differentiation trajectory in limbal basal epithelium of human cornea.. Ocul. Surf..

[31] Norrick A., Esterlechner J., Niebergall-Roth E. (2021). Process development and safety evaluation of ABCB5+ limbal stem cells as advanced-therapy medicinal product to treat limbal stem cell deficiency.. Stem Cell Res. Ther..

[32] Ruiz M., González S., Bonnet C., Deng S.X. (2022). Extracellular miR-6723-5p could serve as a biomarker of limbal epithelial stem/progenitor cell population.. Biomark. Res..

[33] Yang A., Kaghad M., Wang Y. (1998). p63, a p53 homolog at 3q27-29, encodes multiple products with transactivating, death-inducing, and dominant-negative activities.. Mol. Cell.

[34] Kao W.W.Y. (2020). Keratin expression by corneal and limbal stem cells during development.. Exp. Eye Res..

[35] Watanabe K., Nishida K., Yamato M. (2004). Human limbal epithelium contains side population cells expressing the ATP-binding cassette transporter ABCG2.. FEBS Lett..

[36] Ksander B.R., Kolovou P.E., Wilson B.J. (2014). ABCB5 is a limbal stem cell gene required for corneal development and repair.. Nature.

[37] Matic M., Petrov I.N., Chen S., Wang C., Wolosin J.M., Dimitrijevich S.D. (1997). Stem cells of the corneal epithelium lack connexins and metabolite transfer capacity.. Differentiation.

[38] Hernáez-Moya R., González S., Urkaregi A., Pijoan J.I., Deng S.X., Andollo N. (2020). Expansion of human limbal epithelial stem/progenitor cells using different human sera: A multivariate statistical analysis.. Int. J. Mol. Sci..

[39] Sharma S.M., Fuchsluger T., Ahmad S. (2012). Comparative analysis of human-derived feeder layers with 3T3 fibroblasts for the ex vivo expansion of human limbal and oral epithelium.. Stem Cell Rev..

[40] López-Paniagua M., Nieto-Miguel T., de la Mata A. (2016). Comparison of functional limbal epithelial stem cell isolation methods.. Exp. Eye Res..

[41] Chen S.Y., Han B., Zhu Y.T. (2015). HC-HA/PTX3 purified from amniotic membrane promotes BMP signaling in limbal niche cells to maintain quiescence of limbal epithelial progenitor/stem cells.. Stem Cells.

[42] Brejchova K., Trosan P., Studeny P. (2018). Characterization and comparison of human limbal explant cultures grown under defined and xeno-free conditions.. Exp. Eye Res..

[43] de la Mata A., Mateos-Timoneda M.A., Nieto-Miguel T. (2019). Poly-l/dl-lactic acid films functionalized with collagen IV as carrier substrata for corneal epithelial stem cells.. Colloids Surf. B Biointerfaces.

[44] Goldberg M.F. (2013). In vivo confocal microscopy and diagnosis of limbal stem cell deficiency. Photographing the palisades of vogt and limbal stem cells.. Am. J. Ophthalmol..

[45] Schlötzer-Schrehardt U., Dietrich T., Saito K. (2007). Characterization of extracellular matrix components in the limbal epithelial stem cell compartment.. Exp. Eye Res..

[46] Francoz M., Karamoko I., Baudouin C., Labbé A. (2011). Ocular surface epithelial thickness evaluation with spectral-domain optical coherence tomography.. Invest. Ophthalmol. Vis. Sci..

[47] Huang M., Wang B., Wan P. (2015). Roles of limbal microvascular net and limbal stroma in regulating maintenance of limbal epithelial stem cells.. Cell Tissue Res..

[48] Kolli S., Bojic S., Ghareeb A.E., Kurzawa-Akanbi M., Figueiredo F.C., Lako M. (2019). The role of nerve growth factor in maintaining proliferative capacity, colony-forming efficiency, and the limbal stem cell phenotype.. Stem Cells.

[49] Le Q., Xu J., Deng S.X. (2018). The diagnosis of limbal stem cell deficiency.. Ocul. Surf..

[50] Deng S.X., Borderie V., Chan C.C. (2019). Global consensus on definition, classification, diagnosis, and staging of limbal stem cell deficiency.. Cornea.

[51] Prabhasawat P., Ekpo P., Uiprasertkul M., Chotikavanich S., Tesavibul N. (2012). Efficacy of cultivated corneal epithelial stem cells for ocular surface reconstruction.. Clin. Ophthalmol..

[52] Cheng J., Zhai H., Wang J., Duan H., Zhou Q. (2017). Long-term outcome of allogeneic cultivated limbal epithelial transplantation for symblepharon caused by severe ocular burns.. BMC Ophthalmol..

[53] Yazdanpanah G., Haq Z., Kang K., Jabbehdari S., Rosenblatt M., Djalilian A.R. (2019). Strategies for reconstructing the limbal stem cell niche.. Ocul. Surf..

[54] Rama P., Matuska S., Paganoni G., Spinelli A., De Luca M., Pellegrini G. (2010). Limbal stem-cell therapy and long-term corneal regeneration.. N. Engl. J. Med..

[55] Altshuler A., Amitai-Lange A., Tarazi N. (2021). Discrete limbal epithelial stem cell populations mediate corneal homeostasis and wound healing.. Cell Stem Cell.

[56] Saghizadeh M., Kramerov A.A., Svendsen C.N., Ljubimov A.V. (2017). Concise review: Stem cells for corneal wound healing.. Stem Cells.

[57] Li G.G., Zhu Y.T., Xie H.T., Chen S.Y., Tseng S.C.G. (2012). Mesenchymal stem cells derived from human limbal niche cells.. Invest. Ophthalmol. Vis. Sci..

[58] Xie H.T., Chen S.Y., Li G.G., Tseng S.C.G. (2012). Isolation and expansion of human limbal stromal niche cells.. Invest. Ophthalmol. Vis. Sci..

[59] Funderburgh J.L., Funderburgh M.L., Du Y. (2016). Stem cells in the limbal stroma.. Ocul. Surf..

[60] Branch M.J., Hashmani K., Dhillon P., Jones D.R.E., Dua H.S., Hopkinson A. (2012). Mesenchymal stem cells in the human corneal limbal stroma.. Invest. Ophthalmol. Vis. Sci..

[61] Basu S., Hertsenberg A.J., Funderburgh M.L. (2014). Human limbal biopsy–derived stromal stem cells prevent corneal scarring.. Sci. Transl. Med..

[62] Acar U., Pinarli F.A., Acar D.E. (2015). Effect of allogeneic limbal mesenchymal stem cell therapy in corneal healing: role of administration route.. Ophthalmic Res..

[63] Katikireddy K.R., Dana R., Jurkunas U.V. (2014). Differentiation potential of limbal fibroblasts and bone marrow mesenchymal stem cells to corneal epithelial cells.. Stem Cells.

[64] Li G.G., Chen S.Y., Xie H.T., Zhu Y.T., Tseng S.C.G. (2012). Angiogenesis potential of human limbal stromal niche cells.. Invest. Ophthalmol. Vis. Sci..

[65] Kureshi A.K., Dziasko M., Funderburgh J.L., Daniels J.T. (2015). Human corneal stromal stem cells support limbal epithelial cells cultured on RAFT tissue equivalents.. Sci. Rep..

[66] Guo P., Sun H., Zhang Y. (2018). Limbal niche cells are a potent resource of adult mesenchymal progenitors.. J. Cell. Mol. Med..

[67] Zhu H., Wang W., Tan Y. (2022). Limbal niche cells and three-dimensional matrigel-induced dedifferentiation of mature corneal epithelial cells.. Invest. Ophthalmol. Vis. Sci..

[68] Li G., Zhang Y., Cai S. (2018). Human limbal niche cells are a powerful regenerative source for the prevention of limbal stem cell deficiency in a rabbit model.. Sci. Rep..

[69] Komai Y., Ushiki T. (1991). The three-dimensional organization of collagen fibrils in the human cornea and sclera.. Invest. Ophthalmol. Vis. Sci..

[70] Morgan S.R., Dooley E.P., Kamma-Lorger C., Funderburgh J.L., Funderburgh M.L., Meek K.M. (2016). Early wound healing of laser in situ keratomileusis–like flaps after treatment with human corneal stromal stem cells.. J. Cataract Refract. Surg..

[71] Hertsenberg A.J., Shojaati G., Funderburgh M.L., Mann M.M., Du Y., Funderburgh J.L. (2017). Corneal stromal stem cells reduce corneal scarring by mediating neutrophil infiltration after wounding.. PLoS One.

[72] Polisetti N., Schlötzer-Schrehardt U., Reinhard T., Schlunck G. (2020). Isolation and enrichment of melanocytes from human corneal limbus using CD117 (c-Kit) as selection marker.. Sci. Rep..

[73] Higa K., Shimmura S., Miyashita H., Shimazaki J., Tsubota K. (2005). Melanocytes in the corneal limbus interact with K19-positive basal epithelial cells.. Exp. Eye Res..

[74] Nordlund J J (2007). The melanocyte and the epidermal melanin unit: an expanded concept.

[75] Polisetti N., Gießl A., Zenkel M. (2021). Melanocytes as emerging key players in niche regulation of limbal epithelial stem cells.. Ocul. Surf..

[76] Polisetti N., Gießl A., Li S., Sorokin L., Kruse F.E., Schlötzer-Schrehardt U. (2020). Laminin-511-E8 promotes efficient in vitro expansion of human limbal melanocytes.. Sci. Rep..

[77] Polisetti N., Reinhard T., Schlunck G. (2023). Efficient isolation and expansion of limbal melanocytes for tissue engineering.. Int. J. Mol. Sci..

[78] Dziasko M.A., Armer H.E., Levis H.J., Shortt A.J., Tuft S., Daniels J.T. (2014). Localisation of epithelial cells capable of holoclone formation in vitro and direct interaction with stromal cells in the native human limbal crypt.. PLoS One.

[79] Bessou-Touya S., Pain C., Taïeb A., Picardo M., Maresca V., Surlève-Bazeille J-E. (1998). Chimeric human epidermal reconstructs to study the role of melanocytes and keratinocytes in pigmentation and photoprotection.. J. Invest. Dermatol..

[80] Liu L., Nielsen F.M., Emmersen J. (2018). Pigmentation is associated with stemness hierarchy of progenitor cells within cultured limbal epithelial cells.. Stem Cells.

[81] Dziasko M.A., Tuft S.J., Daniels J.T. (2015). Limbal melanocytes support limbal epithelial stem cells in 2D and 3D microenvironments.. Exp. Eye Res..

[82] Pellegrini G., Dellambra E., Golisano O. (2001). p63 identifies keratinocyte stem cells.. Proc. Natl. Acad. Sci. USA.

[83] de Paiva C.S., Chen Z., Corrales R.M., Pflugfelder S.C., Li D.Q. (2005). ABCG2 transporter identifies a population of clonogenic human limbal epithelial cells.. Stem Cells.

[84] Zhao B., Allinson S.L., Ma A., Bentley A.J., Martin F.L., Fullwood N.J. (2008). Targeted cornea limbal stem/progenitor cell transfection in an organ culture model.. Invest. Ophthalmol. Vis. Sci..

[85] Larouche D., Lavoie A., Paquet C., Simard-Bisson C., Germain L. (2010). Identification of epithelial stem cells in vivo and in vitro using keratin 19 and BrdU.. Methods Mol. Biol..

[86] Su G., Guo X., Xu L. (2024). Isolation and characterization of rabbit limbal niche cells.. Exp. Eye Res..

[87] Xie H.T., Chen S.Y., Li G.G., Tseng S.C.G. (2011). Limbal epithelial stem/progenitor cells attract stromal niche cells by SDF-1/CXCR4 signaling to prevent differentiation.. Stem Cells.

[88] Tseng S.C.G., Chen S.Y., Mead O.G., Tighe S. (2020). Niche regulation of limbal epithelial stem cells: HC-HA/PTX3 as surrogate matrix niche.. Exp. Eye Res..

[89] Veréb Z., Póliska S., Albert R. (2016). Role of human corneal stroma-derived mesenchymal-like stem cells in corneal immunity and wound healing.. Sci. Rep..

[90] Tang J., Li Q., Cheng B., Jing L. (2014). Primary culture of human face skin melanocytes for the study of hyperpigmentation.. Cytotechnology.

[91] Pittelkow M.R., Shipley G.D. (1989). Serum‐free culture of normal human melanocytes: Growth kinetics and growth factor requirements.. J. Cell. Physiol..

[92] Willemsen M., Luiten R.M., Teunissen M.B.M. (2020). Instant isolation of highly purified human melanocytes from freshly prepared epidermal cell suspensions.. Pigment Cell Melanoma Res..

[93] Li S., Zenkel M., Kruse F.E., Gießl A., Schlötzer-Schrehardt U. (2022). Identification, isolation, and characterization of melanocyte precursor cells in the human limbal stroma.. Int. J. Mol. Sci..

[94] Hayashi R., Yamato M., Sugiyama H. (2007). N-Cadherin is expressed by putative stem/progenitor cells and melanocytes in the human limbal epithelial stem cell niche.. Stem Cells.

[95] Li J., Chen S.Y., Zhao X.Y., Zhang M.C., Xie H.T. (2017). Rat limbal niche cells prevent epithelial stem/progenitor cells from differentiation and proliferation by inhibiting notch signaling pathway in vitro.. Invest. Ophthalmol. Vis. Sci..

[96] González S., Uhm H., Deng S.X. (2019). Notch inhibition prevents differentiation of human limbal stem/progenitor cells in vitro.. Sci. Rep..

[97] Han B., Chen S.Y., Zhu Y.T., Tseng S.C.G. (2014). Integration of BMP/Wnt signaling to control clonal growth of limbal epithelial progenitor cells by niche cells.. Stem Cell Res. (Amst.).

[98] Su Z., Wang J., Lai Q., Zhao H., Hou L. (2020). KIT ligand produced by limbal niche cells under control of SOX10 maintains limbal epithelial stem cell survival by activating the KIT/AKT signalling pathway.. J. Cell. Mol. Med..

[99] Suanno G., Genna V.G., Maurizi E., Dieh A.A., Griffith M., Ferrari G. (2024). Cell therapy in the cornea: The emerging role of microenvironment.. Prog. Retin. Eye Res..

[100] Dziasko M.A., Daniels J.T. (2016). Anatomical features and cell-cell interactions in the human limbal epithelial stem cell niche.. Ocul. Surf..

[101] Bonnet C., González S., Roberts J.S. (2021). Human limbal epithelial stem cell regulation, bioengineering and function.. Prog. Retin. Eye Res..

[102] Le Q., Chauhan T., Cordova D., Tseng C.H., Deng S.X. (2022). Biomarkers of in vivo limbal stem cell function.. Ocul. Surf..

[103] Bonnet C., Gonzalez S., Deng S.X. (2024). Limbal stem cell therapy.. Curr. Opin. Ophthalmol..

[104] Elhusseiny A.M., Soleimani M., Eleiwa T.K. (2022). Current and emerging therapies for limbal stem cell deficiency.. Stem Cells Transl. Med..

[105] Frucht-Pery J., Siganos C.S., Solomon A., Scheman L., Brautbar C., Zauberman H. (1998). Limbal cell autograft transplantation for severe ocular surface disorders.. Graefes Arch. Clin. Exp. Ophthalmol..

[106] Fogla R., Padmanabhan P. (2005). Deep anterior lamellar keratoplasty combined with autologous limbal stem cell transplantation in unilateral severe chemical injury.. Cornea.

[107] Yalçindag F.N., Incel O., Ozdemir O. (2008). Effectiveness of tacrolimus in high-risk limbal allo-graft transplantation.. Ann. Ophthalmol. (Skokie).

[108] Li J.Y., Cortina M.S., Greiner M.A. (2024). Outcomes and complications of limbal stem cell allograft transplantation.. Ophthalmology.

[109] Pellegrini G., Traverso C.E., Franzi A.T., Zingirian M., Cancedda R., De Luca M. (1997). Long-term restoration of damaged corneal surfaces with autologous cultivated corneal epithelium.. Lancet.

[110] Jurkunas U., Johns L., Armant M. (2022). Cultivated autologous limbal epithelial cell transplantation: New frontier in the treatment of limbal stem cell deficiency.. Am. J. Ophthalmol..

[111] Calonge M., Pérez I., Galindo S. (2019). A proof-of-concept clinical trial using mesenchymal stem cells for the treatment of corneal epithelial stem cell deficiency.. Transl. Res..

[112] Alió del Barrio J.L., De la Mata A., De Miguel M.P. (2022). Corneal regeneration using adipose-derived mesenchymal stem cells.. Cells.

[113] Galindo S., de la Mata A., López-Paniagua M. (2021). Subconjunctival injection of mesenchymal stem cells for corneal failure due to limbal stem cell deficiency: State of the art.. Stem Cell Res. Ther..

[114] Pietryga K., Jesse K., Drzyzga R. (2024). Bio-printing method as a novel approach to obtain a fibrin scaffold settled by limbal epithelial cells for corneal regeneration.. Sci. Rep..

[115] Gurdal M., Ercan G., Barut Selver O., Aberdam D., Zeugolis D.I. (2024). Development of biomimetic substrates for limbal epithelial stem cells using collagen-based films, hyaluronic acid, immortalized cells, and macromolecular crowding.. Life.

